# An American Text-Book of Surgery, for Practitioners and Students

**Published:** 1901-03

**Authors:** 


AN AMERICAN TEXT-BOOK OF SURGERY, for Practitioners and Students, by Phineas S. Connor, M. D., Frederic S. Dennis, M. D., William W. Keene, M. D. Chas. B. Nancrede, M. D., Roswell Park, M. D., Francis J. Shepherd, M. D., Lewis A. Stimpson, M. D., J. Collin Warren, M. D., and J. William White, M. D. Edited by William W. Keene, M. D., LL. D., and J. William White, M. D., Ph. D. Third Edition. Thoroughly Revised. Pp. 1228; Price, $7.00. Philadelphia: W. B. Saunders, 925 Walnut St., 1900.

This work has been before the profession long enough to command the highest testimonials as a standard authority on general surgery. The men who got this book out are leaders in surgery in America. A glance at the list is a sufficient guaranty to the profession.

